# Mechanisms of Allergen-Antibody Interaction of Cockroach Allergen Bla g 2 with Monoclonal Antibodies That Inhibit IgE Antibody Binding

**DOI:** 10.1371/journal.pone.0022223

**Published:** 2011-07-15

**Authors:** Jill Glesner, Sabina Wünschmann, Mi Li, Alla Gustchina, Alexander Wlodawer, Martin Himly, Martin D. Chapman, Anna Pomés

**Affiliations:** 1 INDOOR Biotechnologies, Inc., Charlottesville, Virginia, United States of America; 2 Basic Research Program, SAIC-Frederick, Frederick, Maryland, United States of America; 3 Macromolecular Crystallography Laboratory, National Cancer Institute, Frederick, Maryland, United States of America; 4 Division of Allergy and Immunology, Department of Molecular Biology, University of Salzburg, Salzburg, Austria; King's College, London, United Kingdom

## Abstract

**Background:**

Cockroach allergy is strongly associated with asthma, and involves the production of IgE antibodies against inhaled allergens. Reports of conformational epitopes on inhaled allergens are limited. The conformational epitopes for two specific monoclonal antibodies (mAb) that interfere with IgE antibody binding were identified by X-ray crystallography on opposite sites of the quasi-symmetrical cockroach allergen Bla g 2.

**Methodology/Principal Findings:**

Mutational analysis of selected residues in both epitopes was performed based on the X-ray crystal structures of the allergen with mAb Fab/Fab′ fragments, to investigate the structural basis of allergen-antibody interactions. The epitopes of Bla g 2 for the mAb 7C11 or 4C3 were mutated, and the mutants were analyzed by SDS-PAGE, circular dichroism, and/or mass spectrometry. Mutants were tested for mAb and IgE antibody binding by ELISA and fluorescent multiplex array. Single or multiple mutations of five residues from both epitopes resulted in almost complete loss of mAb binding, without affecting the overall folding of the allergen. Preventing glycosylation by mutation N268Q reduced IgE binding, indicating a role of carbohydrates in the interaction. Cation-π interactions, as well as electrostatic and hydrophobic interactions, were important for mAb and IgE antibody binding. Quantitative differences in the effects of mutations on IgE antibody binding were observed, suggesting heterogeneity in epitope recognition among cockroach allergic patients.

**Conclusions/Significance:**

Analysis by site-directed mutagenesis of epitopes identified by X-ray crystallography revealed an overlap between monoclonal and IgE antibody binding sites and provided insight into the B cell repertoire to Bla g 2 and the mechanisms of allergen-antibody recognition, including involvement of carbohydrates.

## Introduction

Exposure and sensitization to cockroach is associated with the development of asthma, and up to 81% of children in inner-city areas of the U.S. are sensitized to cockroach allergens [1]–[3]. Among such allergens, Bla g 2 is of particular importance, eliciting IgE responses in 58–70% of cockroach allergic patients [4]–[6]. The X-ray crystal structure of Bla g 2 shows a bilobal fold typical of pepsin-like aspartic proteases, but Bla g 2 is enzymatically inactive due to amino acid substitutions in the catalytic site [7], [8]. The structures of both lobes are similar despite the low degree of the amino acid sequence homology (<15% identity) [9]. The presence of a zinc binding site and five disulfide bridges in Bla g 2 adds stability to the protein, thus favoring persistence of the allergen in the environment [7]. Chronic exposure to low doses (<1 µg/g dust) of this stable cockroach allergen may explain the association between sensitization to Bla g 2 and asthma [3], [6].

Most reports on epitope mapping of allergens focus on the identification of linear epitopes by using libraries of overlapping synthetic peptides, recombinant fragments of the allergen, epitope expression cDNA libraries, or digested allergens. These approaches are especially useful for food allergens where linear epitopes are common due to allergen digestion (in addition to conformational epitopes) [10], [11]. However, conformational IgE antibody binding epitopes are important for inhaled allergens which reach the respiratory system mostly in their original globular structure. Little is known about the IgE antibody binding repertoire, the clonality and affinity of the interactions, the location and structure of epitopes and the kind of interactions involved in antibody recognition of the allergen [12]–[15]. Our aim was to map conformational antigenic determinants of Bla g 2 using the tertiary structure of the molecule as a “template” for mutagenesis, and to analyze the effect of amino acid substitutions on mAb and IgE antibody binding in order to gain insight on the IgE antibody binding repertoire. The epitope mapping approach presented here was based on the determination of the molecular structure of two allergen-antibody complexes by X-ray crystallography, which provided an accurate molecular structure of the conformational epitopes. Subsequently, site-directed mutagenesis was performed to confirm that the amino acids found at the interfaces are essential for antibody binding. For mapping B cell epitopes, it is not feasible to co-crystallize IgE antibodies with the allergen, due to the polyclonal nature of IgE and its paucity in sera (<1 µg/ml compared with approximately 10 mg/ml for IgG). Therefore, mAb 7C11 and 4C3 which interfere with the binding of IgE antibodies, were selected to facilitate the identification of residues and kinds of interactions involved in such binding.

Fragments of the non-overlapping mAb 7C11 and 4C3, raised against natural Bla g 2, were independently co-crystallized with recombinant Bla g 2 (rBla g 2) and the structures were determined [16], [17]. Subsequent site-directed mutagenesis of the residues involved in the mAb epitopes led to the identification of amino acids that are important for the allergen-mAb interaction. Additionally, amino acids involved in IgE binding were identified, indicating an overlap of epitopes for IgE and monoclonal antibodies.

## Materials and Methods

### Sera from cockroach allergic patients

Sera from cockroach allergic patients were obtained from commercial sources (Bioreclamation, Inc., Westbury, NY) or from stored samples that had been collected from patients enrolled in 1988–1989 in Wilmington (Delaware) and Charlottesville (Virginia) for epidemiological studies performed at the University of Virginia [18], [19]. Bioreclamation operates in full compliance with Food and Drug Administration guidelines. The studies performed at the University of Virginia were approved by the Human Investigation Committee and blood was drawn after informed written consent was obtained from the patient. IgE antibody levels in sera ranged from 7 to 640 ng of total IgE/ml (average 147±177 ng/ml) [18] and 0.4–100 ng of IgE against Bla g 2/ml (average 41±27 ng/ml), measured by mAb-based RIA [4]. A pool of 6 sera from cockroach allergic patients highly sensitized to Bla g 2 was used for inhibition assays. The pool contained 18.6 kU_A_/L of cockroach extract-specific IgE (i6) and 4.2 kU_A_/L of Bla g 2-specific IgE by ImmunoCAP (Phadia AB, Uppsala, Sweden) measured by using streptavidin CAPs coated with biotinylated Bla g 2.

### Rational design of mutagenesis

Rational design of site-directed mutagenesis was based on the crystal structures of Bla g 2 alone and in complex with the fragments of the mAb 7C11 and 4C3 (PDB accession codes 1yg9, 2nr6 and 3liz, respectively) [7], [16], [17]. Structural images were created with the program PyMol [20].

### Site-directed-mutagenesis

Five rBla g 2 mutants, each containing a single amino acid substitution within the 7C11 binding epitope (K65A, D68aA, R83A, E86A, and K132A), as well as eight mutants of the epitope for the mAb 4C3 (E233A, D248A, K251A, E233R, and E233R-I199W on N93Q; E233R and E233R-I199W on N268Q, and N268Q alone) were designed. The mutations E233A, D248A, K251A, E233R, and E233R-I199W were performed on the rBla g 2-N93Q mutant which is the molecule that had been crystallized (alone and in complex with mAb fragments) and served as the basis for the mutational analysis. The N93Q substitution, distant from the mAb epitopes, had been introduced in order to prevent glycosylation of Bla g 2, which interferes with crystallization. Thus the rBla g 2-N93Q was considered to contain the intact mAb epitopes. In addition, mutants E233R and E233R-I199W with a N268Q mutation (instead of N93Q), and the N268Q mutation alone, were also designed. The Bla g 2-DNA was inserted into the yeast *P. pastoris* expression vector pGAPZαC for constitutive expression of the allergen. Site-directed mutagenesis was performed using QuikChange™ (Stratagene). Sequence of the mutated DNA was confirmed before linearization and transformation into the *P. pastoris* strain KM71.

### Expression, purification, and analysis of Bla g 2 mutants in *Pichia pastoris*

Expression of recombinant proteins in *P. pastoris* was performed as described [8]. Recombinant Bla g 2-N93Q (with intact epitopes for 7C11 and 4C3 mAbs), rBla g 2-N268Q, and epitope mutants were purified from culture media by affinity chromatography using either mAb 7C11 or 4C3. Both monoclonals had been produced against natural Bla g 2, and recognize both natural and the recombinant allergen. Parallel dose-response curves were observed for both recombinant and natural Bla g 2 by two-side mAb binding ELISAs using combinations of 7C11 and 4C3 mAb.

Analysis of recombinant mutants by circular dichroism (CD) and mass spectrometry was performed as follows. The far UV CD spectra were recorded at concentrations around 0.1 mg/ml upon addition of 10 mM sodium phosphate pH 7.4 between 190 and 260 nm, using a Jasco J-810 spectropolarimeter (Japan Spectroscopic), temperature-controlled at 20°C. NanoLC-MSMS-based peptide mapping (CapLC, Micromass-Waters) of proteolytic digests was performed using trypsin and V8 protease (Roche). For the R83A mutant, additional digests were mapped with subtilisin and pepsin (Sigma-Aldrich). MSMS data were evaluated automatically using PLGS 2.2.5™ software. An *in house* allergen sequence databank was used for MSMS data search in nonspecific digestion mode with carbamidomethylation set as fixed modification.

### Inhibition of IgE antibody binding by mAb 7C11

Microplates were coated with 20 µg/ml rBla g 2-N93Q in 50 mM carbonate-bicarbonate buffer, pH 9.6. The antibodies tested for inhibition of IgE antibody binding were the anti-Bla g 2 mAb 7C11 (at 0, 0.001, 0.01, 0.1, 1 and 10 µg/ml concentrations), and a rabbit polyclonal anti-Bla g 2 antibody (at 1∶10 dilutions). These antibodies were added, followed by addition of sera from cockroach allergic patients with high anti-Bla g 2 IgE antibody titer (1∶4 or 1∶10 final dilution). Incubation was performed at room temperature for 2 h. Affinity purified peroxidase labeled goat anti-human antibody IgE (Kirkegaard and Perry Laboratories) was used for detection at 1∶1000. Plates were developed as previously described [21].

### ELISA to measure rBla g 2 mutants and dose-response curves of epitope mutants

Recombinant Bla g 2 was measured by ELISA using either the mAb 7C11 or 4C3 as capture antibody and a Bla g 2-specific rabbit polyclonal antibody (pAb) for detection. A standard containing natural Bla g 2 was used starting at 250 ng/ml.

The epitope mutants and rBla g 2-N93Q were compared by performing dose-response experiments using mAb 7C11 or 4C3 as capture antibodies and biotinylated mAb 4C3 or 7C11 for detection, respectively, or polyclonal antibody. The biotinylated and polyclonal antibodies used for capture were first titrated in order to select the concentration to be used in the dose-response experiments. This concentration was the lowest possible, closest to the Kd of the allergen-antibody interaction (nM range for mAb 7C11 and 4C3; 8 nM for mAb 4C3-Fab; Dr. Sandra Smith-Gill and Claudia Lipschultz, personal communication), while still providing a window of antibody binding activity (OD 405 nm = 1–2). The concentrations of biotinylated antibodies were 4 nM and 21 nM for mAb 7C11 (1∶500,000) and 4C3 (1∶100,000), respectively, and 53 nM for the polyclonal antibody (1∶100,000).

### Inhibition of IgE and monoclonal antibody binding by epitope mutants

Inhibition ELISA were performed to compare the mutants of the epitopes for mAb 7C11 and 4C3 for their capacity to inhibit binding of the respective antibody to rBla g 2. In brief, mutants and rBla g 2-N93Q were preincubated in tubes for 1 h at 4°C with biotin-labeled mAb (at 1∶5000 dilution; 580 nM and 428 nM for biotinylated mAb 7C11 and 4C3, respectively) or IgE antibodies (sera pool diluted 1∶2) and then added to microtiter plates coated with 10 µg/ml rBla g 2-N93Q. After 1 h (3 h for IgE antibodies) incubation, plates were washed and incubated for 30 min with streptavidin-peroxidase, and developed as above. The allergens selected for testing inhibition of IgE antibody binding to rBla g 2-N93Q were: Bla g 2-N93Q, Bla g 2-N268Q, natural Bla g 2, K65A, D68aA, K132A, E233R, E233R-I199, E233R-I199-N268Q and K251A.

### Multiplex fluorescent array

Monoclonal antibodies 7C11 and 4C3 (20 µg) were coupled to two different Luminex carboxylated fluorescent microsphere bead sets (Luminex Corp., Austin, TX) and the multiplex fluorescent array was performed as described [22], [23]. The mAb coupled beads were added along with rBla g 2-N93Q or mutants at a concentration of 400 ng/ml. The mutants of the epitope for the mAb 7C11 were tested using beads coated with the mAb 4C3, and *vice versa*. Sera (1∶5 dilution) or anti-Bla g 2 rabbit pAb (1∶1,000 dilution) were added to wells, mixed with beads, and incubated. The concentration of IgE in sera is in nM range and was diluted further for the assay. Biotin labeled goat anti-human IgE (KPL, Gaithersburg, MD) or biotin labeled goat anti-rabbit IgG (Jackson ImmunoResearch, West Grove, PA), both at 1∶1000 dilution, were added to the wells that had been incubated with sera or anti-Bla g 2 rabbit pAb, respectively. Biotinylated mAbs were added to the wells where a mAb sandwich assay was performed. In the final step, streptavidin-phycoerythrin (4 µg/ml) was added to all wells and mixed. Absorbance was measured in a Bio-Plex fluorescent suspension array reader (Bio-Rad Laboratories, Hercules, CA). Each mutant was compared to rBla g 2-N93Q by paired, Student t-test. P values lower than 0.05 were considered significant.

## Results

### Inhibition of IgE antibody binding by mAb 7C11

**Figure 1:**
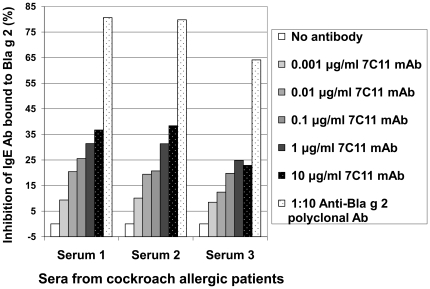
Overlap of 7C11 mAb and IgE antibody epitopes. Inhibition of IgE antibody binding to Bla g 2 by mAb 7C11 (0.001–10 µg/ml) and a rabbit anti-Bla g 2 polyclonal antibody.

Increasing concentrations of mAb 7C11 (from 0.001 to 10 µg/ml) showed dose dependent inhibition of IgE antibody binding of up to 40% (Figure 1). Additional 3 sera showed an average inhibition of 25%. The rabbit anti-Bla g 2 polyclonal antibody showed inhibition of IgE antibody binding of up to 82%. Background inhibition of IgE antibody binding by mAb 4C1 against mite-allergen Der p 1 was observed below 7% at 100 µg/ml (data not shown). Inhibition of IgE antibody binding by mAb 4C3 was previously reported [17].

### Comparison of IgE antibody binding to Bla g 2 presented by either 7C11 or 4C3 monoclonal antibodies

IgE and rabbit polyclonal antibody binding to Bla g 2 presented by mAb 7C11 or 4C3 was compared using mAb coupled microspheres in a multiplex array. A monomeric Bla g 2 mutant was used as the capture allergen to avoid the possible influence of dimerization of Bla g 2 in determining levels of antibody binding [16]. The amino acid substitutions in the monomeric mutant do not involve residues from the mAb epitopes and thus do not prevent binding of both mAbs.

**Figure 2:**
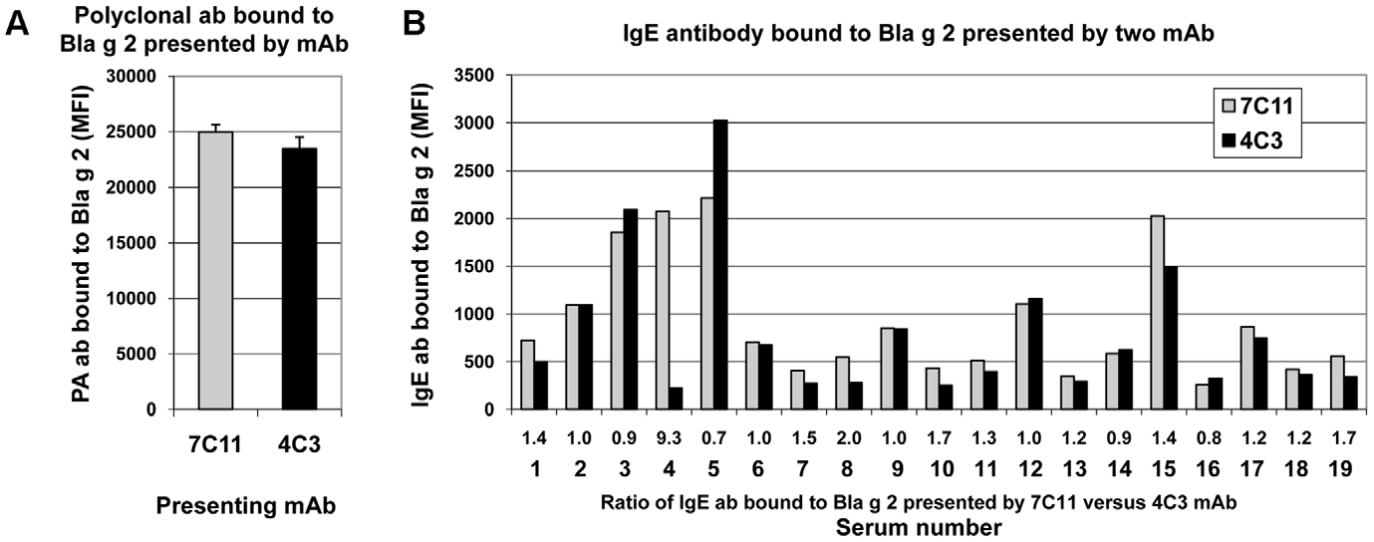
Antibody binding to rBla g 2 presented by mAb 7C11 or 4C3 using multiplex array. A) Anti-Bla g 2 polyclonal antibody bound to rBla g 2 (mean ± standard deviation, n = 5 experiments, p = 0.059). B) IgE from sera of cockroach allergic patients bound to rBla g 2. The allergen was presented by either mAb 7C11 (gray) or 4C3 (black). The first and second row of numbers under the X-axis are ratio of IgE bound to Bla g 2 presented by mAb 7C11 versus mAb 4C3 (100%) and serum number, respectively. MFI are Median Fluorescence Intensity Units.

Polyclonal anti-Bla g 2 antibody binding to the allergen monomer was very similar for the allergen presented by either mAb 7C11 or 4C3 (Figure 2A). Forty nine sera from cockroach allergic patients were tested and 19 sera with Median Fluorescence Intensity Units (MFI)>200 were chosen for further analysis. The amount of IgE antibody bound to the allergen presented by either mAb varied by 10-fold, from 200 to >3000 MFI (Figure 2B). In general, similar amounts of IgE antibody were bound to rBla g 2 presented by either mAb 7C11 or 4C3 (ratios close to 1 in Figure 2B), indicating that there was not an immunodominant lobe of Bla g 2 for most patients.

### Rational design of Bla g 2 epitope mutants based on crystal structures of allergen-antibody complexes

**Figure 3:**
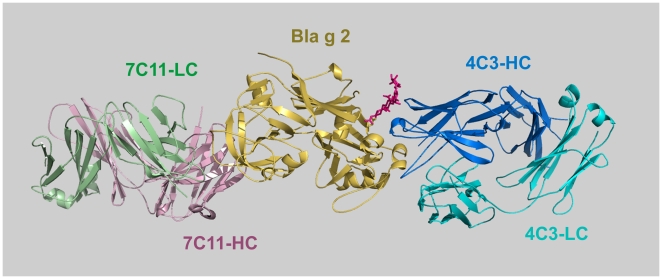
Epitopes for mAb 7C11 and 4C3 are located on opposite lobes of Bla g 2. Ribbon diagram of rBla g 2 (gold) in complex with a Fab fragment of the mAb 4C3 (blue) superimposed to the Fab′ of mAb 7C11 in complex with Bla g 2 (allergen not shown). Monoclonal Ab 7C11 binds to the N-terminal lobe of the allergen and mAb 4C3 to the C-terminal lobe. Antibody fragments consist of a heavy and a light chain, in green and light lilac for 7C11, and in marine blue and cyan for 4C3, respectively. The carbohydrates in position N268 are shown in red.

**Figure 4:**
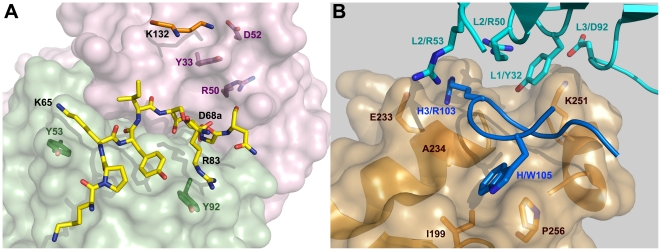
Rational design for mutagenesis. A) Principal residues involved in the the mAb 7C11 epitope on the N-terminal lobe of Bla g 2. The longest consecutive part of the epitope (shown in yellow) interacts with all three CDR of the light chain (surface in green) and has 9 residues (starting at 60) from which two were mutated to alanine (K65 and D68a). K65, R83 and K132 form cation-π interactions with tyrosines Y53, Y92 and Y33 from the mAb 7C11, respectively. The heavy chain of the antibody is shown in lilac. B) Main residues involved in the mAb 4C3 epitope on the C-terminal lobe of Bla g 2. The surface of the allergen, with the main residues involved in antibody binding is shown in gold. The third loop of the heavy chain of the antibody (in blue) includes: a) R103 attracted to the negatively charged E233, and b) W105 that docks into a hydrophobic pocked formed by A234, I199 and P256 in Bla g 2. Residues K251 and Y32 involved in a cation-π interaction are also shown.

The relevance of specific amino acids for 7C11 and 4C3 mAb binding was investigated by site-directed mutagenesis of selected residues in the epitopes identified by X-ray crystallography which are located on the N and C-terminal lobes of Bla g 2, respectively (Figure 3). They interact with complementarity determining regions (CDRs) of the heavy (H1, H2, H3) and light (L1, L2, L3) chains of the antibody [16], [17]. Regarding the epitope for the mAb 7C11, residues K65 and D68a were selected as part of the longest loop which, together with R83, primarily interacts with the light chain of the antibody (Figure 4A, in yellow). Residue D68a is unique in the allergen since it interacts with both heavy and light chains. Residues R83, E86 and K132 which interact with the CDRs L3, H3 and H1/H2, respectively, were also mutated. E86, as well as D100, maintain the orientation of the side chain of R101-H3 by forming electrostatic interactions with this arginine, which is also involved in multiple hydrophobic interactions with I67 from the long loop of the epitope. K132 forms strong hydrogen bonds with D52-H2 and additional contacts with residues from H1. Three of the five residues selected for mutagenesis (K65, R83 and K132) form cation-π interactions with tyrosines 53-L2, 92-L3 and 33-H1 from the antibody (Figure 4A).

In contrast to the epitope for the mAb 7C11, interactions of the mAb 4C3 with the allergen involve carbohydrates and solvent molecules (water and zinc), in addition to amino acids, and the CDR L2 is not involved in the interaction [17]. Mutagenesis aimed to disrupt two main anchoring sites of the mAb 4C3. One site involves amino acids from three loops of the allergen (199–202, the long loop 248–259 and 268–273) plus residues from the C-terminal end of the helix 225–238. The most specific interactions are between the positively charged R103 from CDR H3 of the mAb 4C3 and the negatively charged C-terminal end of the allergen helix 225–235, enhanced by the presence of a negatively charged E233 (Figure 4B). Mutation of E233 to either a neutral alanine or a positively charged arginine was aimed at disrupting these charge-charge interactions. Residue I199 was also mutated to the bulkier tryptophan in order to prevent the docking of W105 from the CDR H3 into a hydrophobic pocket on Bla g 2 formed by A234, I199, and P256 (Figure 4B). Aspartate 248 interacts with metal (most likely Zn^2+^) in one of the two observed conformations and was mutated to alanine. This mutation aimed to disrupt an extensive hydrogen bonded network formed by water molecules and metal that mediates the remaining contacts between CDRs L1 and L3 and two loops of the allergen (involving residues 248–252 and A234). Finally, lysine 251, which is the only residue interacting directly with the CDRs L1 and L3 of the antibody through Y32 (forming a cation-π interaction) and D92, respectively, was mutated to alanine (Figure 4B).

Another anchoring site of mAb 4C3 is a carbohydrate at position N268, whose trimannosyl core interacts with CDRs H1 and H2, plus N74 from the third loop of the heavy chain of the antibody (usually not involved in recognition) (Figure 3). The mutant N268Q was expressed alone to prevent glycosylation at this site. In addition, mutants E233R and E233R-I199W were expressed with and without N268Q to disrupt the interaction of mAb 4C3 with amino acids and carbohydrates simultaneously. In summary, this study is a mutagenesis analysis of the 7C11 mAb epitope and an extension to our previously reported analysis of the 4C3 mAb epitope which examined only the effect of substituting three amino acids on monoclonal antibody binding [17].

### Analysis of monoclonal antibody binding to the epitope mutants

**Figure 5:**
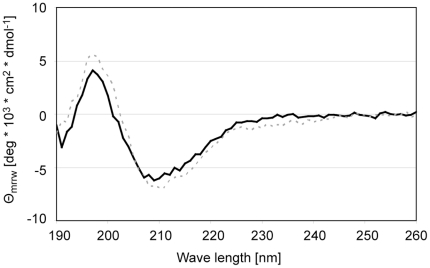
CD spectra of natural Bla g 2 (black line) and rBla g 2-N93Q (gray dashed).

**Figure 6:**
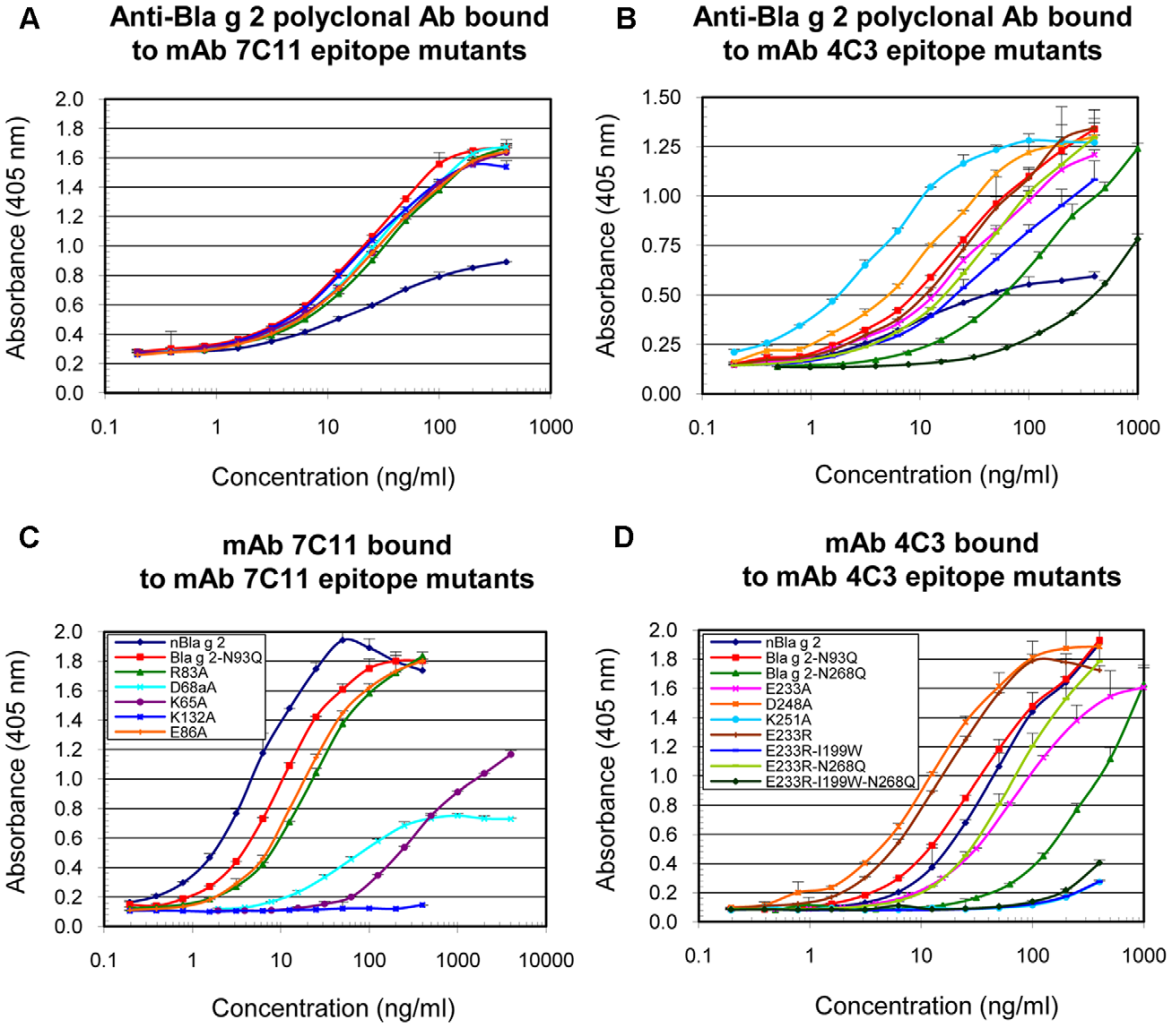
Dose-response curves of Bla g 2 and epitope mutants by ELISA. A) Binding of the anti-Bla g 2 polyclonal antibody to mAb 7C11 epitope mutants. B) Binding of the anti-Bla g 2 polyclonal antibody to mAb 4C3 epitope mutants. C) Binding of mAb 7C11 to mAb 7C11 epitope mutants, D) Binding of mAb 4C3 to mAb 4C3 epitope mutants. Data are mean ± standard deviation of duplicates from one representative experiment out of two performed for each panel. Legends in panels C and D also apply to panels A and B, respectively.

The overall folding of all the purified rBla g 2 mutants was shown to be similar to the rBla g 2-N93Q. Purified rBla g 2-N93Q and epitope mutants showed a main band at ∼37 kDa (monomer) and a minor band at ∼74 kD (dimer) as expected on SDS-PAGE, under non-reducing conditions [4]. Mutants of the epitope for the mAb 7C11 were subjected to extensive quality control by mass spectrometry and circular dichroism. The mutations were confirmed by mass spectrometry. CD spectra of the mutants of the epitope for the mAb 7C11, K65A, R83A, E86A and K132A, were comparable to rBla g 2-N93Q and natural Bla g 2, with minima between 205 and 215 nm and maxima below 200 nm, indicating similar overall folding (Figure 5). Only the D68aA CD spectrum displayed a higher β-sheet content, as visualized by a negative shoulder between 220 and 230 nm (data not shown). Additional antibody binding assays confirmed an overall proper folding of the mutants: when presented by mAb coupled to microspheres, they showed similar binding of a polyclonal Bla g 2-specific antibody, with parallel dose-response curves (Figure 6A). These results indicated that the mutated allergens had preserved conformational epitope(s) required for mAb capture and for binding of polyclonal antibodies, showing that they were overall properly folded. Subsequently, the control for correct overall folding of mutated allergens was performed by measuring binding of a Bla g 2-specific polyclonal antibody to the mutants presented by the mAb that binds to the lobe opposite to the mutation. Results showed that the overall folding of the mutants of the epitope for the mAb 4C3 was also similar to rBla g 2-N93Q (Figure 6B). In contrast with the mAb 7C11 epitope mutants, the dose-response curves for certain mAb 4C3 epitope mutants were displaced to the right, indicating an effect of these mutations (especially the N268Q affecting glycosylation) on the polyclonal antibody binding (Figure 6B).

Binding of the polyclonal antibody to recombinant Bla g 2 resulted in a wider window of absorbance compared to the natural allergen (Figures 6A and B), whereas the same window was obtained for natural and recombinant allergen with the two combinations of mAb (Figure 6C and D). This could be explained by the fact that the polyclonal antibody was raised against recombinant Bla g 2, and although the natural and recombinant allergens have similar CD spectra (Figure 5), differences in epitopes recognized by the polyclonal antibody (maybe involving different carbohydrates) could occur.

### A) Monoclonal antibody binding to the mAb 7C11 epitope mutants

Parallel dose-response curves were obtained for natural Bla g 2, rBla g 2-N93Q and the epitope mutants E86A, R83A and K65A. The strongest effect on reduction of mAb 7C11 binding resulted from the mutation K132A (Figure 6C). The three curves for E86A, R83A and K65A were displaced to the right compared to rBla g 2-N93Q, indicating a lower binding affinity of these mutants for mAb 7C11, whereas the mutant D68aA lost its parallelism with rBla g 2-N93Q.

In agreement with the dose-response curves by ELISA, and as controls for the IgE antibody binding experiments performed by multiplex array, a reduction of mAb 7C11 binding to four out of five mutants of the epitope for the mAb 7C11 (K65A, D68aA, R83A and K132A) presented by mAb 4C3 coupled to microspheres was observed by multiplex technology, whereas the same level of polyclonal antibody binding was obtained at these assay conditions (data not shown). Lower levels of mAb binding to the epitope mutants compared to rBla g 2-N93Q (except for E86) were also observed in multiplex dose-response curves using 25 to 800 ng/ml of allergen, whereas the levels of bound anti-Bla g 2 polyclonal used as detection antibody were equivalent (data not shown).

**Figure 7:**
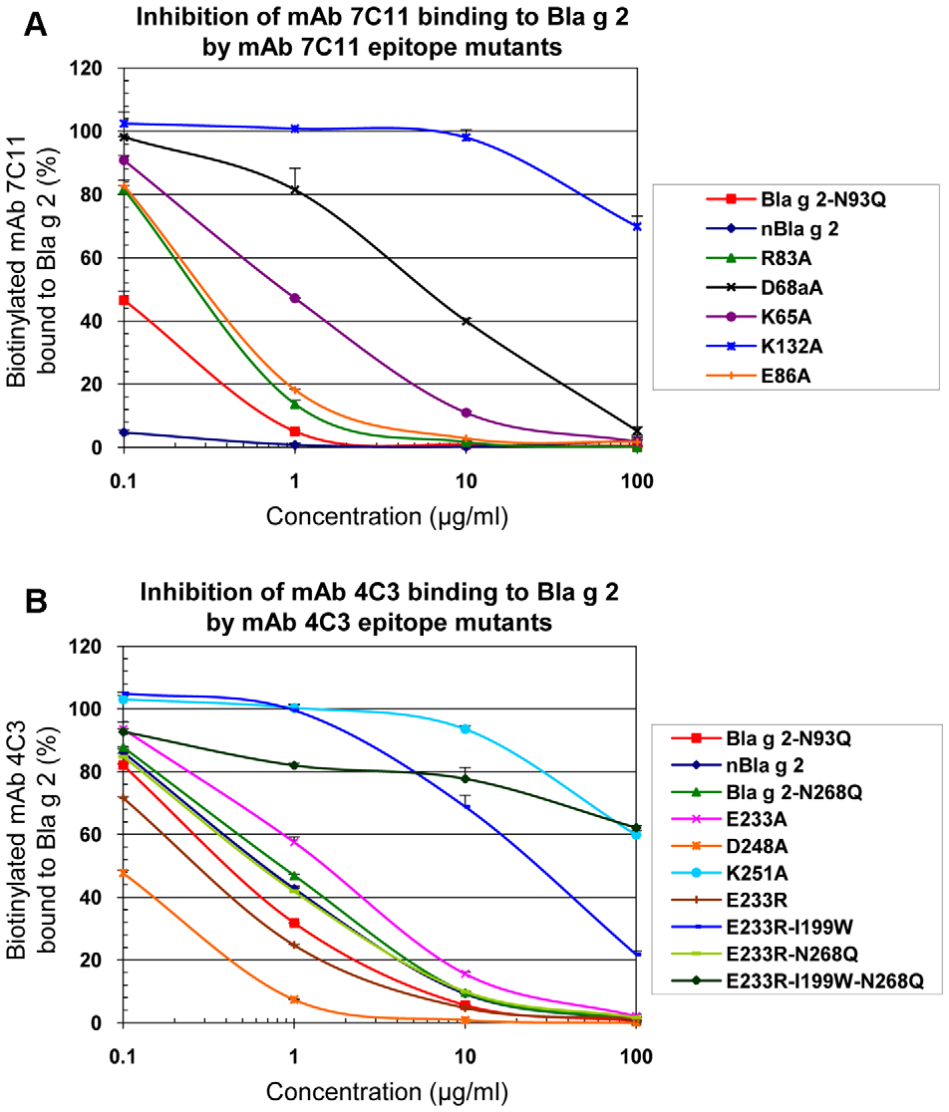
Inhibition of biotinylated mAb binding to rBla g 2 by allergen mutants using ELISA. A) Inhibition of binding of biotinylated-mAb 7C11 to rBla g 2-N93Q by mAb 7C11 epitope mutants. B) Inhibition of binding of biotinylated-mAb 4C3 to rBla g 2-N93Q by mAb 4C3 epitope mutants. Data are mean ± standard deviation of duplicates from one representative experiment out of two performed for each panel.

As a validation test, the dose-response curves and multiplex array results were also compared to competition ELISA to evaluate the ability of the mutants to compete with solid phase-bound rBla g 2-N93Q for binding to the mAbs (Figure 7A). The order of potency of the mutants for inhibiting mAb 7C11 binding to Bla g 2 was: natural Bla g 2>Bla g 2-N93Q>R83A∼E86A>K65A>D68aA>K132A, in agreement with the relative binding affinities obtained from the dose-response curves (Figure 6C). In particular, mutant K132A did not inhibit binding of mAb 7C11 to rBla g 2-N93Q at 10 µg/ml, dose at which the other mutants showed significant or total levels of inhibition (Figure 7A). This result shows an important loss in binding affinity of K132A for mAb 7C11, in agreement with ELISA and multiplex results.

### B) Monoclonal antibody binding to the mAb 4C3 epitope mutants

The mutants of the epitope for the mAb 4C3 showed similar binding to mAb 7C11 by ELISA, but had either similar or reduced binding to mAb 4C3 compared to rBla g 2-N93Q. The strongest effect on reduction of mAb 4C3 resulted from the mutations K251A and E233R-I99W (with or without N268Q) (Figure 6D). Dose-response curves showed a displacement to the right for most of the mutations affecting the mAb 4C3 epitope, except for D248A and E233R. The order of potency of the N93Q mutants for inhibiting mAb 4C3 binding to Bla g 2 was: E233R>Bla g 2-N93Q>E233R-I199W, similarly to the N268Q mutants: E233R-N268Q>Bla g 2-N268Q>E233R-I199W-N268Q. Combination of the mutations I199W with E233R had a strong effect on reducing mAb 4C3 (Figure 6D and 7B). ELISA dose-response curves of the N268Q rBla g 2 mutants that lack carbohydrates at that position were displaced to the right compared to the equivalent allergens non mutated at position 268 (i.e. E233-N268 and E233-I199-N268 versus E233 and E233-I199, respectively) similarly to what was observed for the enzymatically deglycosylated allergen [17]. Once the multiplex assays were validated with all the mutants by ELISA dose-response curves and inhibition assays, the IgE antibody binding assays were performed by multiplex array technology in order to reduce the amount of the sera used for the assays.

### Analysis of IgE antibody binding to rBla g 2 mutants in a fluorescent multiplex array assay

**Figure 8:**
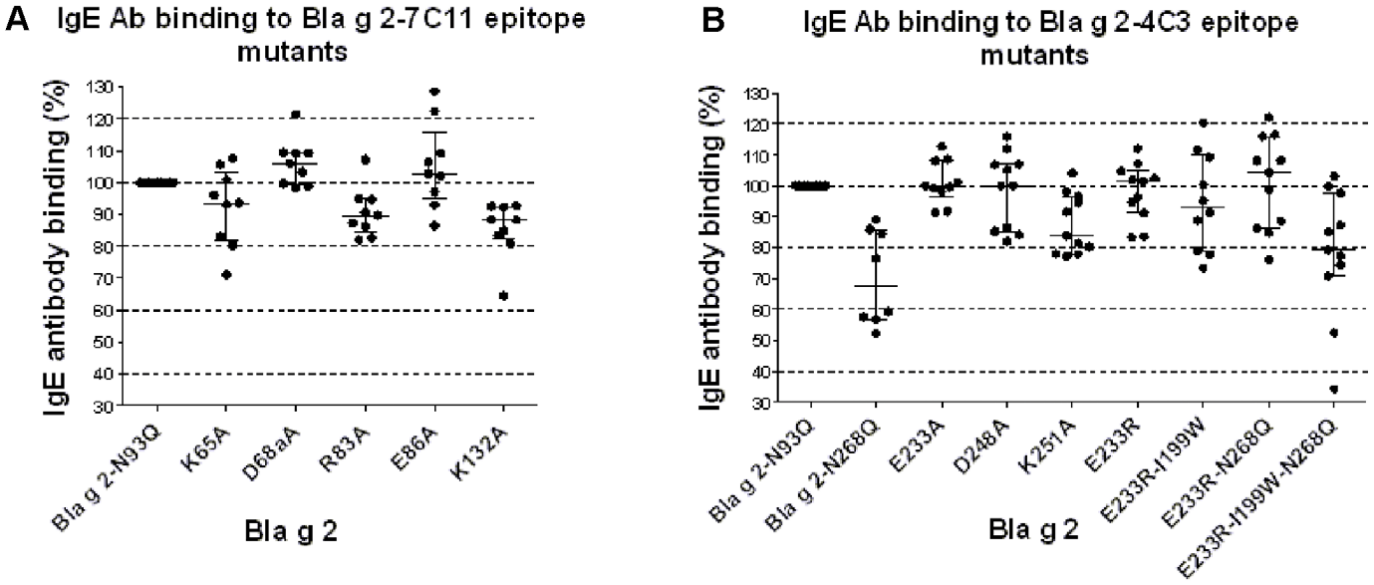
Binding of IgE antibodies from cockroach allergic patients to rBla g 2-N93Q and epitope mutants. A) IgE binding to mAb 7C11 epitope mutants, and B) IgE binding to mAb 4C3 epitope mutants. In A and B scatter plots show median of percentages (± interquartile ranges) of IgE binding versus rBla g 2-N93Q for the sera tested (n = 8–11) Each data point was calculated as average of percentage of IgE binding (± SEM) versus the rBla g 2-N93Q for 1–4 tests per serum depending on serum availability.

The effects of mutations on IgE antibody binding varied by patient. The mutations that led to lowest IgE antibody binding for certain sera were K65A, K132A, N268Q and E233R-I199W-N268Q (Figure 8). There was no effect on IgE antibody binding for certain mutations and some of the patients. The concentration of IgE in sera is in the nM range (i.e. estimated 65 nM for the sera pool), and should be most likely low enough to allow changes in antibody binding to be visualized.

Despite inter-patient differences, the reduction in the percentage of IgE bound to each mutant versus the rBla g 2-N93Q was statistically significant for K65A, R83A, K132A, Bla g 2-N268Q, K251A and E233R-I199W-N268Q. In inhibition assays using a pool of six sera from patients highly sensitized to Bla g 2, the mutants that had less capacity to inhibit IgE antibody binding to rBla g 2 were E233R-199W-N268Q, followed by K251A (data not shown).

**Figure 9:**
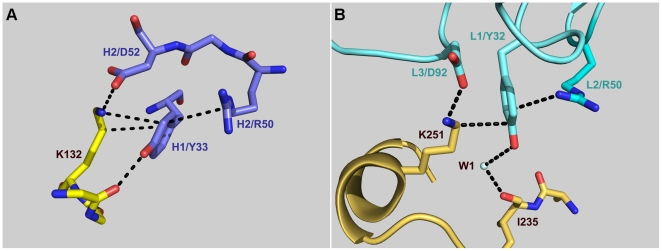
Structural environment of the lysine residues involved in cation-π interactions important for antibody binding. A) Lysine 132 interacting with the tyrosine 33 of the heavy chain of the mAb 7C11. B) Lysine 251 interacting with the tyrosine 32 of the light chain of the mAb 4C3.

Mutations K132A and K251A affected cation-π interactions between allergen and antibody. The sum of percentage of decrease in IgE antibody binding for the two mutants K132A and K251A versus rBla g 2-N93Q ranged from 7 to 51% with an average of 25% (n = 9 sera). The interaction between each lysine and the mAb showed a very similar structure (Figure 9): lysines 132 and 251 establish cation-π interactions with tyrosines that are supported by a hydrogen bond with R50 from the heavy and light chains of the antibodies 7C11 and 4C3, respectively. The two lysine residues also interact with D52 and D92 from the heavy and light chains of the antibodies, respectively.

## Discussion

Bla g 2 is a major inhalant allergen associated with cockroach sensitization and the development of asthma. We have previously reported the crystal structures of Bla g 2 alone [7] and in complex with Fab′ of the mAb 7C11 and Fab of the mAb 4C3 [16], [17]. Both mAb inhibit IgE antibody binding up to 40–45%, as shown here for 7C11 and previously for 4C3 [17]. Taking advantage of the fact that both mAb bind to opposite lobes of Bla g 2, we compared mAb and IgE antibody binding to mutants of the epitopes presented by the mAb that binds on the opposite site of the mutation. This experimental approach had the advantage of analyzing antibody binding to non-fragmented allergen and conformational epitopes. In contrast, most previous epitope mapping studies reported patient differences in IgE antibody epitope recognition by measuring antibody binding to fragments or peptides of an allergen which have lost the structural environment required for conformational epitopes [10], [11].

A rational design of site-directed mutagenesis was performed based on the three-dimensional structure of both mAb epitopes [16], [17]. Mutagenesis analysis showed that most of the amino acids selected from different loops of the conformational epitopes were contributing to the allergen-mAb interactions. Non effective mutations either affected only one of the few interactions of the allergen with a single residue of the antibody (E86), or an indirect interaction through a metal ion (D248). The most important residues were K132 for the mAb 7C11 epitope and I199 and K251 for the mAb 4C3 epitope. These mutations led to a) a displacement to the right of dose-response curves compared to curves of allergens with non-mutated epitope, and b) a decrease of the relative inhibitory potency of the mutant for mAb binding to Bla g 2, whereas the overall folding of the allergen was preserved. Therefore, the decrease in antibody binding could be directly attributed to local changes due to the amino acid substitution and was not caused by overall structural alterations or breakdown of the molecule. Interestingly, four of the effective mutations on mAb binding (K65A, R83A, K132A, and K251A) disrupted cation-π interactions and proved their importance for protein-protein recognition [24], [25]. The two most important ones (involving K132 and K251) had a strikingly similar structural environment. The relevance of lysine and positively charged amino acids in allergen-IgE antibody interactions has previously been reported [26]–[30]. In these studies, the rationale for selection of residues to mutate was based on sequence or structural alignments, the identification of solvent accessible residues (i.e. lysine in high amounts on the allergen surface), or on epitopes previously identified by testing IgE antibody binding to synthetic peptides corresponding to the allergen sequence by immunoblotting or dot-spots [26]–[30]. In the present study, the structural environment and mechanisms of allergen-mAb interaction are revealed by a detailed analysis of the structure of allergen-antibody complexes solved by X-ray crystallography.

Binding of mAb 4C3 to Bla g 2 was reduced by mutations aimed at: a) the disruption of charge-charge interactions (E233A), and, b) the disruption of antibody binding to a hydrophobic pocket on Bla g 2 (I199W). The addition of the I199W substitution to the E233R mutants further decreased antibody binding (as evidenced by a displacement of the dose-response curves to the right) showing the importance of the CDR3-H for the antibody interaction. This region has been reported to be the most variable in length, sequence and structure and sufficient for most antibody specificities [31], [32].

Selected mutations from a rational design based on the crystal structures of allergen-antibody complexes strongly affected the binding of the mAb (K132A, K251A, E233R-I199W and E233R-I199W-N268Q). However, such an important effect in reducing antibody binding was not observed for IgE antibody binding, as expected from mutating amino acids from only one of the multiple epitopes involved in the IgE antibody response. Single substitutions of lysines or arginines forming cation-π interactions resulted in a decrease of IgE antibody binding. There was a tendency for a stronger reduction of IgE antibody binding for mutants containing the I199W substitution, as observed for monoclonal antibody binding. These IgE antibody binding reductions are small but detectable since other substitutions of similar residues (E, R, K) in other parts of the molecular surface did not show this effect, with medians of IgE antibody binding around 100% and narrower interquartile ranges (between 98 and 111%) (data not shown). Single point allergen mutants have previously been shown not to lead to significant reduction of IgE reactivity [29], [33]. These results indicate that the IgE antibody response to Bla g 2 does not involve a single immunodominant epitope. In contrast to a large number of studies for mapping linear epitopes on allergens, only one previous study followed a similar approach for indirectly identifying IgE antibody binding sites on Bet v 1 using a murine mAb that inhibits IgE antibody binding [34]. In this study, the Bet v 1 residue E45 was reported to be critical for a major IgE antibody binding epitope. Mutation of E45 to serine decreased IgE antibody binding up to 60%, indicating that this epitope was immunodominant, although not unique in the IgE antibody repertoire [34].

We observed a contribution of sugars to the Bla g 2 interaction with mAb 4C3 antibody by a displacement to the right of allergen dose-response curves for the N268Q mutants (single substitution or in combination with either E233R or E233R-I199W) versus the equivalent allergen mutants glycosylated at this position 268. We previously reported similar results with an enzymatically deglycosylated rBla g 2 [17]. The 4C3 mAb epitope is a good model for this analysis because, to the best of our knowledge, it is the only epitope that involves interaction with both amino acids and carbohydrates [17]. In this study, the contribution of carbohydrates on IgE antibody binding to Bla g 2 is shown as well. Carbohydrates at position 268 appeared to be part of a “double anchorage” of the IgE antibody, involving carbohydrates and amino acids. First, removal of sugars by the N268Q substitution resulted in a significant reduction of IgE antibody binding compared to rBla g 2-N93Q. Second, a significant reduction of the IgE antibody binding to the mutant E233R-I199A was only observed when the N268Q substitution was also present. These results indicate that disruption of the interaction by amino acid substitution combined with removal of carbohydrate interfered with the two anchorage sites for IgE. In summary, these results further support the existence of common IgE epitopes among cockroach allergic patients which overlap with mAb epitopes, and prove the involvement of carbohydrates in IgE antibody binding to Bla g 2. The importance of carbohydrates on IgE antibody binding and their relevance on allergic disease is a matter of debate. Cross-reactive carbohydrate determinants (CCD) from plants and insects are N-glycans recognized by IgE, with minimal or no clinical significance [35]–[37]. In contrast, recent data suggest that IgE antibodies to mammalian carbohydrate epitopes can be an important factor in anaphylaxis. Reactions to the mAb cetuximab and delayed reactions to red meat are related to serum IgE antibodies against oligosaccharide galactose-α-1,3-galactose (alpha-gal), as a consequence of tick bites [38]. Although reduction of IgE antibody binding to de-glycosylated allergens has been previously reported, this study shows the mechanisms (kind of interactions and residues) and the structural elements (trymanosyl core of the N-glycans) involved in the interaction with mAb 4C3, and how removal of carbohydrates at position 268 impairs IgE antibody binding. Further studies on the effect of carbohydrates attached to Bla g 2 at the T cell level and on the induction of mediator-release are underway.

Interestingly, the relative importance of a mutated epitope versus the remaining epitopes involved in the IgE response varied by patient. For specific patients, the IgE antibody binding to some of the mutants was reduced by 35–65%, indicating that the mutated epitope was important for the allergic response of these individual patients. These results are consistent with the fact that the degree of IgE antibody binding inhibition by monoclonal antibodies also varies depending on the sera. Similarly, small percentages of patients (10–25%) with >50% reduction of IgE antibody binding to specific single mutants has previously been reported for Blo t 5 [29]. Differences in the decrease of antibody binding by patient and mutation further supports heterogeneity in the IgE antibody repertoire among allergic patients. Heterogeneity in epitope recognition has also been observed for other allergens [26], [28], [29], [33].

Despite differences in IgE antibody binding by patient and per mutation, the overall pattern of reduction of IgE antibody binding was not random. Mutations that affected the binding of IgE antibody to one patient also tended to reduce the binding for other patients, while other mutations had little effect on antibody binding. This is most obvious for the 7C11 mAb epitope mutants K65A, R83A and K132A which showed a reduced binding of IgE from most subjects (with the most pronounced effect for K132A), whereas mutations D68aA and E86A did not show this effect. A similar reduction of IgE antibody binding was also seen for the 4C3 mAb epitope mutants K251A and N268Q. The surface on Bla g 2 covered by a mAb (893 Å^2^ in average) only represents ∼6% of the total surface of the allergen (15,061 Å^2^). The relatively larger reduction of binding of specific IgE for some sera caused by a single or multiple amino acid change on the molecular surface of Bla g 2 further emphasizes that the selected residues are important for IgE antibody binding. Reduction in either affinity of IgE antibody binding to a specific mutated epitope or in clonality (if the binding to the epitope is abolished by the mutation) are possible reasons for the reduction of IgE antibody binding observed. These two properties of the IgE antibody repertoire, affinity and clonality, have been reported to determine the effector cell degranulation in response to allergen challenge [12]. The interest of the study also relies on devising methods to reduce the IgE binding capacity of allergens without disrupting their overall structure, and to increase the knowledge of which residues in an epitope can be mutated for the greatest effect on antibody binding.

In summary, the epitopes for mAb 7C11 and 4C3 are important surface determinants overlapping with IgE antibody binding sites. The epitope mapping approach involved a three-dimensional analysis of epitopes whose structure had been solved by X-ray crystallography. Therefore, mechanisms of allergen-antibody interaction, including carbohydrates as part of the epitope or cation-π interactions, could be elucidated. Key amino acids for antibody binding were also identified, in contrast to previous studies that predicted potential recognition sites according to specific properties of amino acids from the allergen (their relevant surface, electrostatic properties) and/or considering if these properties were different in human homologues. This study is part of a larger body of work to better understand conformational epitopes on allergens for design of immunotherapy vaccines with reduced IgE antibody binding. In addition, results provided insight on the polyclonal IgE antibody response, regarding differences in the IgE epitope repertoire among individuals. The possible existence of immunodominant epitopes and the relative importance of IgE antibody binding sites were investigated. Overall, this study demonstrates that the crystal structure of allergen-Ab complexes can be used for detailed epitope mapping of conformational epitopes, to identify the most important residues for antibody binding and to elucidate mechanisms of allergen-antibody interaction.
